# Supplementary material for: Effects of BPA Exposure and Recovery on the Expression of Genes Involved in the Hepatic Lipid Metabolism in Male Mice
Source: Toxics. 2023 Sep 12;11(9):775. doi: 10.3390/toxics11090775 (PMC10535508; doi:10.3390/toxics11090775)
Supplement: Supplementary file 1 [file toxics-11-00775-s001.zip › toxics-2525462-supplementary.pdf]

Supporting Information

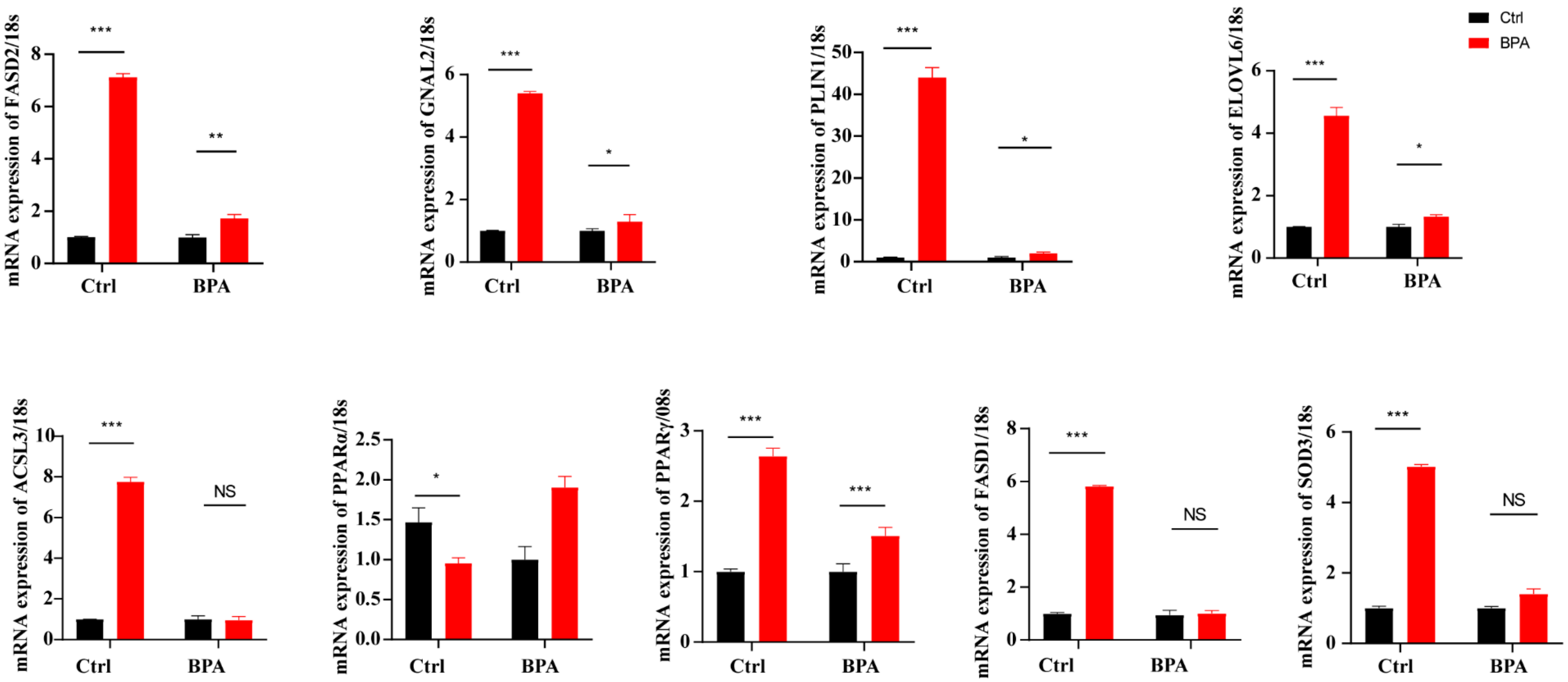

Figure S1. Effects of BPA exposure on the expression of key genes in liver.

*Represents the significance at $p < 0.05$. **Represents the significance at $p < 0.01$. *** Represents the significance at $p < 0.001$.

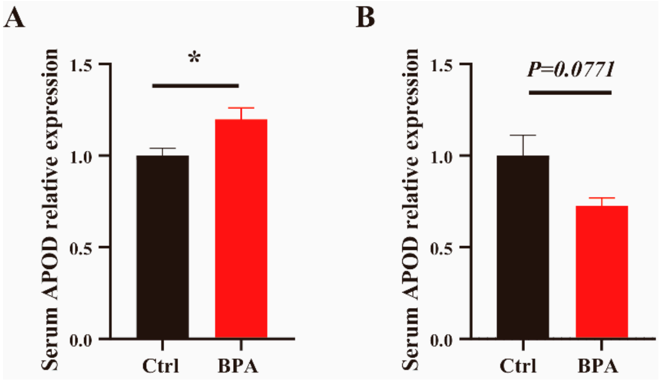

Figure S2. serum APOD levels between comparison groups

Note: (A) APOD levels for 8 weeks; (B) APOD levels for 16 weeks. *Represents the significance at $p < 0.05$.

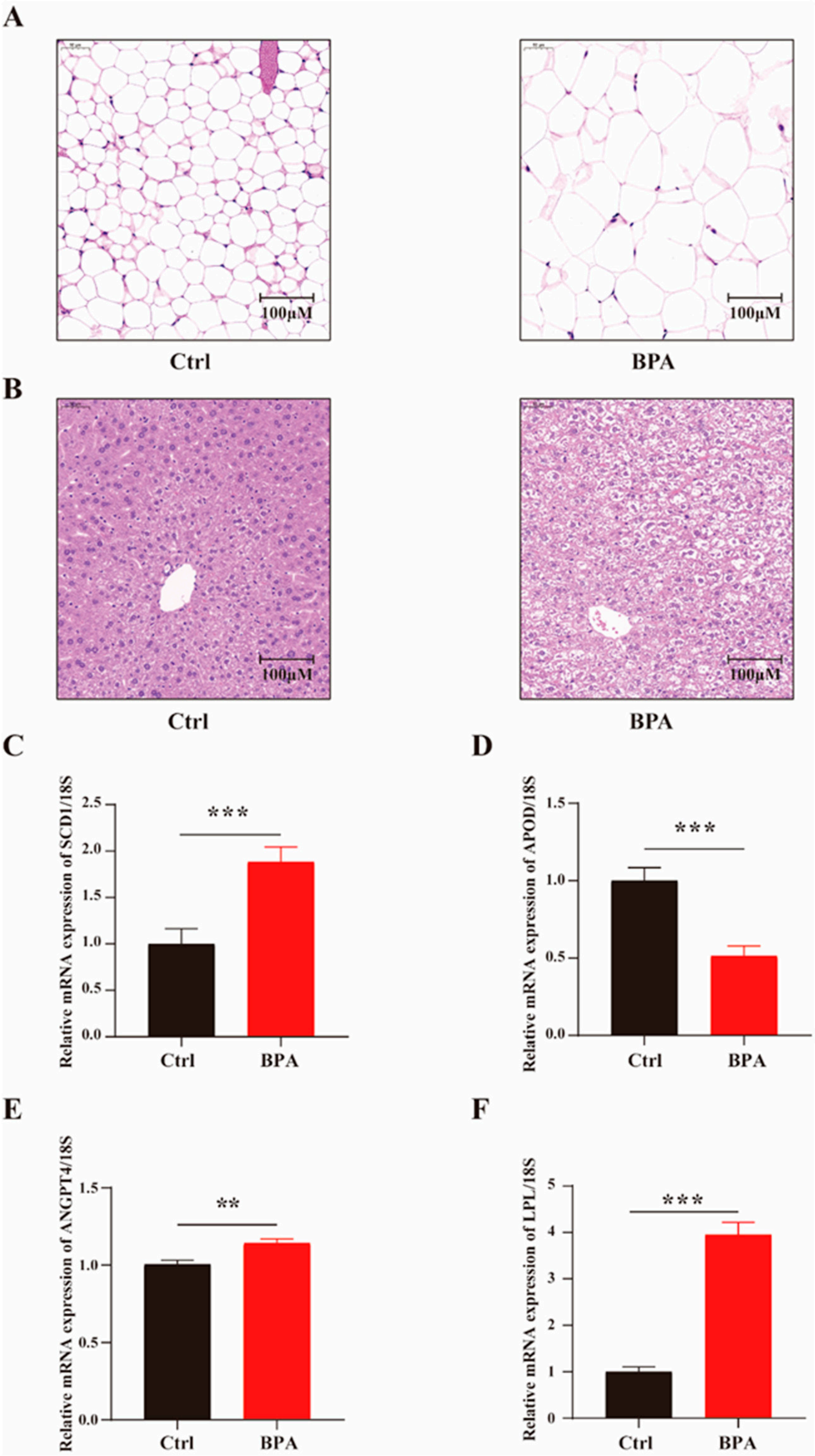

Figure S3. Effects of 16 weeks of continuous BPA exposure on mice phenotype

Note: (A) Representative images of adipose tissue stained with H&E in mice; (B) Representative images of liver with H&E in mice; (C)-(F) Effects of BPA on the hepatic mRNA expression of genes regulating lipid metabolism. n=5 in each group; Statistical significance was determined by one-way ANOVA. **Represents the significance at $p < 0.01$. *** Represents the significance at $p < 0.001$.

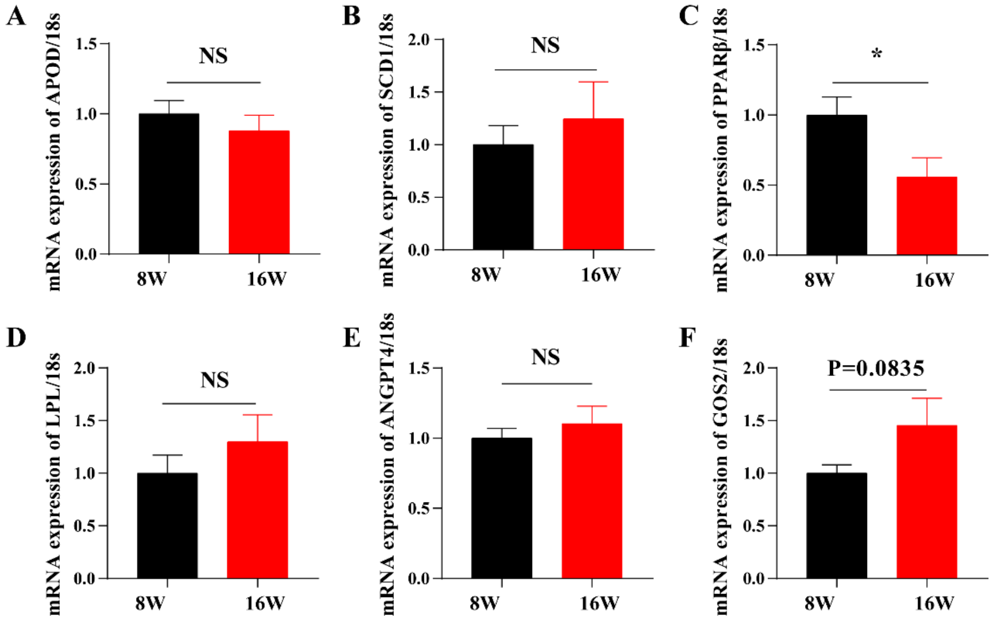

Figure S4. Effects of age factors on the expression of genes in the mice liver

Note: Statistical significance was determined by one-way ANOVA. *Represents the significance at $p < 0.05$.

Table S1. PCR primers for qRT-PCR validation

Gene		Primers(5'to3')	Products(bp)
ACSL3(mouse)	F	GCGAGAAGGATTCCAAGACTGG	125
	R	GAAGAGTAGCCGATTCGGCATC	
ANGPT4(mouse)	F	TACAGCGCCTGGTACGGATTGT	94
	R	CGCTTGATCTCTGCACAGTCCT	
ApoD(mouse)	F	TGAACCAAGTAAAGGGTGAAGC	134
	R	CACGAGGGCATAGTTTTCAT	
ELOVL6(mouse)	F	CGGCATCTGATGAACAAGCGAG	120
	R	GTACAGCATGTAAGCACCAGTTC	
FADS1(mouse)	F	ACCTGTCAGTCTTTGGCACCTC	139
	R	TCCTTGCGGAAGCAGTTAGGCT	
FADS2(mouse)	F	TTCCTGGAGAGCCACTGGTTTG	132
	R	GAAGAAGGACTGCTCCACATTGC	
GNAI2(mouse)	F	CAGATCGACTTTGCTGATCCC	191
	R	TAAGCGGCTGAGTCATTGAGC	
G0S2(mouse)	F	GCTAGTGAAGCTATACGTGCTGG	157
	R	GGACTGCTGTTCACACGCTTCC	
LPL(mouse)	F	GTAGCAGGAAGTCTGACCAATAAG	116
	R	AAATCAGCGTCATCAGGAGA	
PLIN1(mouse)	F	AAGCCTTGTGAGGAGGGTCA	522
	R	AGGGCATCGGATAGGGACAT	
SCD1(mouse)	F	TGCTGGGGCGAGACTTTTGT	138
	R	GGATTGAATGTTCTTGTCGTAGGG	
SOD3(mouse)	F	GACCTGGTTGAGAAGATAGGCG	124
	R	TGGCTGATGGTTGTACCCTGCA	
PPARα(mouse)	F	ACCACTACGGAGTTCACGCATG	102
	R	GAATCTTGCAGCTCCGATCACAC	
PPARβ(mouse)	F	CGCACCCTTTGTCATCCAC	120
	R	CTGGCAGCGGTAGAACACG	
PPARγ(mouse)	F	CTCCAAGAATACCAAAGTGCGA	335
	R	TCGCACTTTGGTATTCTTGGAG	
β-actin(mouse)	F	CATTGCTGACAGGATGCAGAAGG	138
	R	TGCTGGAAGGTGGACAGTGAGG	
